# Variation in competent and respectful delivery care in Kenya and Malawi: a retrospective analysis of national facility surveys

**DOI:** 10.1111/tmi.13361

**Published:** 2020-01-02

**Authors:** Catherine Arsenault, Mike English, David Gathara, Address Malata, Wilson Mandala, Margaret E. Kruk

**Affiliations:** ^1^ Department of Global Health and Population Harvard T.H. Chan school of Public Health Boston MA USA; ^2^ Kenya Medical Research Institute (KEMRI) ‐ Wellcome Trust Research Programme Nairobi Kenya; ^3^ Nuffield Department of Medicine University of Oxford Oxford UK; ^4^ The Academy of Medical Sciences Malawi University of Science and Technology Thyolo Malawi

**Keywords:** quality, labour, childbirth, health systems research, sub‐Saharan Africa, qualité, travail, accouchement, recherche sur les systèmes de santé, Afrique subsaharienne

## Abstract

**Objective:**

Although substantial progress has been made in increasing access to care during childbirth, reductions in maternal and neonatal mortality have been slower. Poor‐quality care may be to blame. In this study, we measure the quality of labour and delivery services in Kenya and Malawi using data from observations of deliveries and explore factors associated with levels of competent and respectful care.

**Methods:**

We used data from nationally representative health facility assessment surveys. A total of 1100 deliveries in 392 facilities across Kenya and Malawi were observed and quality was assessed using two indices: the quality of the process of intrapartum and immediate postpartum care (QoPIIPC) index and a previously validated index of respectful maternity care. Data from standardised observations of care were analysed using descriptive statistics and multivariable random‐intercept regression models to examine factors associated with variation in quality of care. We also quantified the variance in quality explained by each domain of covariates (patient‐, provider‐ and facility‐level and subnational divisions).

**Results:**

Only 61–66% of basic elements of competent and respectful care were performed. In adjusted models, better‐staffed facilities, private hospitals and morning deliveries were associated with higher levels of competent and respectful care. In Malawi, younger, primipara and HIV‐positive women received higher‐quality care. Quality also differed substantially across regions in Kenya, with a 25 percentage‐point gap between Nairobi and the Coast region. Quality was also higher in higher‐volume facilities and those with caesarean section capacity. Most of the explained variance in quality was due to regions in Kenya and to facility, and patient‐level characteristics in Malawi.

**Conclusions:**

Our findings suggest considerable scope for improvement in quality. Increasing staffing and shifting births to higher‐volume facilities – along with promotion of respectful care in these facilities – should be considered in sub‐Saharan Africa to improve outcomes for mothers and newborns.

## Abstract (fr)

**Objectif:**

Bien que des progrès substantiels aient été accomplis dans l'amélioration de l'accès aux soins pendant l'accouchement, les réductions de la mortalité maternelle et néonatale ont été plus lentes. Des soins de mauvaise qualité peuvent être à blâmer. Dans cette étude, nous mesurons la qualité de la main‐d'œuvre et des services d'accouchement au Kenya et au Malawi en utilisant les données des observations des accouchements et explorons les facteurs associés aux niveaux de la compétence et du respect dans les soins.

**Méthodes:**

Nous avons utilisé les données d'enquêtes d'évaluation des établissements de santé représentatives au niveau national. 1100 accouchements dans 392 établissements au Kenya et au Malawi ont été observés et la qualité a été évaluée à l'aide de deux indices: l'indice de qualité du processus de soins intra‐partum et postpartum immédiat (QoPIIPC) et un indice précédemment validé de soins maternels respectueux. Les données des observations normalisées des soins ont été analysées à l'aide de statistiques descriptives et de modèles de régression à interceptions aléatoires multivariables pour examiner les facteurs associés à la variation de la qualité des soins. Nous avons également quantifié la variance de la qualité expliquée par chaque domaine de covariables (divisions au niveau des patients, des prestataires et des établissements, et infranationales).

**Résultats:**

Seuls 61% à 66% des éléments de base de soins compétents et respectueux ont été réalisés. Dans les modèles ajustés, des établissements mieux dotés en personnel, des hôpitaux privés et des accouchements le matin étaient associés à des niveaux plus élevés de soins compétents et respectueux. Au Malawi, les femmes plus jeunes, primipares et VIH positives ont reçu des soins de meilleure qualité. La qualité différait également considérablement d'une région à l'autre au Kenya, avec un écart de 25 points de pourcentage entre Nairobi et la région côtière. La qualité était également plus élevée dans les établissements avec un volume plus élevé et ceux ayant une capacité de césarienne. La majeure partie des raisons de la variance dans la qualité était liée aux régions du Kenya et à l'établissement et aux caractéristiques des patients au Malawi.

**Conclusions:**

Nos résultats suggèrent une marge considérable pour l'amélioration de la qualité. L'augmentation du personnel et le déplacement des naissances vers des établissements de plus grand volume – ainsi que la promotion de soins respectueux dans ces établissements – devraient être envisagés en Afrique subsaharienne pour améliorer les résultats pour les mères et les nouveau‐nés.

## Introduction

According to the recent Lancet Global Health Commission on quality, high‐quality health systems are characterised by three key domains: strong foundations, good health outcomes and the provision of competent and respectful care 1. While ample data exist on the quality of foundations (i.e. the availability of facilities, electricity, staffing and tools required for care) and on levels of maternal and newborn health outcomes, less is known about levels of competent and respectful care received by mothers and newborns in sub‐Saharan Africa 2, 3, 4.

Despite rising coverage of facility deliveries, an estimated 2.7 million newborns die in the first month of life, and 303 000 women die from causes related to childbirth and pregnancy, more than half of whom live in sub‐Saharan Africa 5, 6. Ensuring competent and respectful care during childbirth is crucial to prevent, detect and treat potential complications, improve maternal and newborn survival, and increase people’s confidence and trust in the health system.

Few studies have examined the quality of the processes of care for individual deliveries, that is the provision of competent and evidence‐based clinical actions during childbirth. Several qualitative studies have pointed to disrespectful treatment of women during childbirth, but there is less quantitative evidence on levels of dis/respectful maternity care in LMICs 7. Identifying factors associated with variation in competent and respectful care may provide a starting point for diagnosing reasons for poor performance and could help identify opportunities for improving quality.

In this paper, we draw from nationally representative service provision assessment (SPA) surveys in Kenya and Malawi, which contain standardised observations of routine delivery services 8, 9. We describe the levels of competent and respectful care and explore potential determinants of variation in quality.

## Methods

### Data sources

We used data from the Service Provision Assessment (SPA) surveys conducted in Kenya in 2010 and in Malawi in 2013–2014. The SPA surveys, developed by the Demographic and Health Surveys Program, have been conducted in several LMICs since the 1990s. The goal of the SPA survey is to provide a comprehensive overview of health service delivery in a country. The SPA surveys include four instruments: a facility audit, interviews with health providers, direct observations of care (family planning consultations, antenatal care, labour and delivery care and sick child care) and exit interviews with patients. We included Kenya and Malawi because they were the only two countries where the SPA survey conducted observations of labour and delivery services. In Kenya, the SPA survey used a randomly selected nationally representative sample of all health facilities. All three national referral hospitals and all eight provincial hospitals in Kenya were included 8. In Malawi, the survey was based on a census of all public health facilities and large private facilities, and on a representative sample of small private facilities 9. In each facility, delivery clients were selected for observation based on the number of women present on the day of the survey. The rule was to observe a maximum of five delivery clients for each provider, with a maximum of 15 deliveries per facility.

### Quality measures

We used two previously validated indices of competent and respectful care to measure quality of labour and delivery care services. Tripathi and colleagues 10 developed an index to assess the quality of the process of intrapartum and immediate postpartum care (QoPIIPC) in facility deliveries. This index is based on 20 process of care indicators available in the SPA related to the initial assessment and examination of the patient, the management of the first, second and third stages of labour and immediate newborn and postpartum care (Figure S1). In this study, we estimate care competence using the QoPIIPC index.

We also used the respectful maternity care index developed and validated by the Maternal and Child Health Integrated Program (MCHIP) 11. This index was based on nine indicators of provider–client interactions reflecting actions the provider should take to ensure the client is informed and able to make choices about her care, and that her dignity and privacy are respected (Figure S1).

### Missing data

In both countries, the labour and delivery observation checklist was divided into four sections: (i) initial client assessment, (ii) care during first stage of labour, (iii) care during birth and (iv) immediate newborn and postpartum care. For several women, the full labour and delivery process could not be observed because the observation began when labour had already started, or because the woman was referred to another facility during the first stage of labour (most of them for caesareans). In both countries, 8–9% of babies needed resuscitation at birth so routine postpartum care was not observed. This systematic missingness precluded the use of multiple imputation. Rather, we decided to calculate the quality index based on the sections observed for each woman. The denominator therefore varied across women. Nonetheless, 73% of women (400 in each country) were observed for all four sections of the labour and delivery process and had no missing data for the 29 quality indicators. As a sensitivity test, we repeated the analyses in this subsample.

### Covariates

We explored potential determinants of competent and respectful care at the patient, provider and facility levels. The covariates considered for inclusion were identified based on prior research suggesting that they may influence quality of care and provider behaviour 1, 12. Availability of these covariates differed between the two countries. In Malawi, several characteristics of the delivering women were available including her age, time of delivery, whether she was HIV positive and whether she was giving birth for the first time. In Kenya, only the time of day during which the delivery took place was available at the patient level. In both countries, provider‐level covariates included gender and cadre and facility‐level covariates included the facility type, whether the facility had the capacity to perform caesarean sections, the ratio of full‐time clinical health professionals (medical and nursing) per maternity bed and the annual volume of deliveries. An indicator for urban location was also available in Malawi. Finally, in both countries, we also included indicator variables for subnational divisions as defined by the SPA surveys: eight regions in Kenya and five zones in Malawi. All covariates were included as binary or categorical variables for better interpretability. Health worker cadres were grouped into two categories based on years of training for country‐specific cadres. Higher cadres included MDs, clinical technicians, medical assistants, BScN and registered nurses and BScN and registered midwives. Lower cadres included enrolled nurses, enrolled midwives, community health nurses and nurse aides. The thresholds for categories of annual volume of deliveries (<500, 500–1500, >1500) were selected to reflect international thresholds for high and low volume facilities. In Kenya, annual volume of deliveries was reported in the survey. Because this variable was not available in the Malawi survey, we estimated annual delivery volume by multiplying the number of delivery clients present in the facility on the day of the survey by 365. Finally, the ratio of clinical staff per maternity bed was divided into quintiles and included in the analysis as a binary indicator comparing the top quintile to all other facilities.

### Statistical analysis

We first explored differences in quality across levels of the covariates by performing pairwise comparisons of means, using the Bonferroni method to adjust for multiple comparisons for categorical indicators. We then constructed multivariable two‐level random‐intercept regression models, with patients nested within providers, and standard errors clustered by facility. All covariates were included in the multivariable models for the exception of caesarean section capacity and annual delivery volumes which were strongly collinear with facility types.

To quantify the variance explained by each domain of covariates (patient, provider, facility and subnational divisions), we progressively added blocks of variables to the multilevel models. We calculated the percentage of variation in quality explained by the group of covariates as the difference in variance between the adjusted model and the null model divided by the null model variance. All regression analyses were performed separately in each country and were not adjusted for sampling weights. The SPA survey used the same methods for observations of care in Kenya and Malawi. The quality indices were therefore measured identically in both countries. However, other questionnaires differed and certain characteristics of women and facilities were only available in one of the two countries. We therefore opted to conduct regression analyses separately by country. However, as a sensitivity analysis, we repeated the regression by pooling data and including covariates available in both countries. We conducted two additional sensitivity analyses. First, we conducted the analyses in the subsample of 800 women with complete data. Second, we performed the regression analyses using patient‐level sampling weights.

All statistical analyses were performed using Stata version 14.2 (Stata Corp, College Station, United States of America). This study was funded by the Bill and Melinda Gates foundation.

### Ethics

The Harvard T.H. Chan School of Public Health institutional review board approved this study as exempt from full review. The corresponding author had full access to all the data in the study and had final responsibility for the decision to submit for publication.

## Results

Table 1 shows the characteristics of the 1100 deliveries observed. In both countries, the majority of deliveries took place in the morning and were attended by female providers. Providers in Kenya had more training on average than those in Malawi: 67% of deliveries were attended by more trained cadres *vs.* only 23% in Malawi. Most women in Malawi were 20 to 35 years old, 70% had already given birth and 5.4% were HIV positive. In both countries, most deliveries took place in public hospitals and in facilities with caesarean section capacity. The 75th percentile of clinical staff per maternity bed was five in Kenya and three in Malawi.

**Table 1:** Characteristics of labour and delivery care observations in Kenya (2010) and Malawi (2013–2014), service provision assessment (SPA) surveys

	Kenya (*N* = 626)		Malawi (*N* = 474)	
	*N* †	%	*N* †	%
Patient				
Evening and night (6 pm–6 am)	85	13.7	26	5.6
Afternoon (12 pm–6 pm)	192	30.8	117	24.6
Morning (7 am–12 pm)	347	55.6	331	69.8
Age 35+			57	12.0
Age 20–35			316	66.7
Age 19 or younger			101	21.4
First childbirth: No			330	69.6
First childbirth: Yes			144	30.4
HIV negative			448	94.6
HIV positive			26	5.4
Provider				
Male	104	16.6	95	20.2
Female	522	83.4	379	79.9
Lower Cadre‡	210	33.5	364	76.8
Higher Cadre§	416	66.5	110	23.2
Facility				
Rural Location			207	43.8
Urban Location			267	56.3
Public health centre	62	9.9	106	22.3
Private health centre	43	6.9	33	7.0
Public hospital	419	66.9	275	58.0
Private hospital	102	16.3	60	12.7
C‐section capacity: No	190	30.3	165	34.9
C‐section capacity: Yes	436	69.7	309	65.1
Annual volume of deliveries ¶ < 500	149	23.7	57	12.0
Annual volume of deliveries 500–1500	183	29.3	114	24.0
Annual volume of deliveries 1501+	294	47.0	303	64.0
Clinical staff/maternity bed†† Lowest four quintiles	459	73.4	390	82.4
Clinical staff/maternity bed Highest quintile	167	26.7	84	17.6
Regions				
Coast	47	7.6		
Central	76	12.1		
Eastern	104	16.6		
Nairobi	59	9.4		
Northeastern	37	5.9		
Nyanza	138	22.1		
Rift Valley	98	15.7		
Western	66	10.5		
Zones				
Central east			99	20.9
Central west			150	31.6
Northern			54	11.3
South east			89	18.7
South west			82	17.4

† Includes patient‐level sampling weights

‡ Lower cadres include enrolled nurses and midwives, community health nurses and nurse aides.

§ Higher cadres include MDs, clinical technicians, medical assistants, BScN nurses and midwives and registered nurses and midwives.

¶ Measured by the number of deliveries reported by the facility in the past 12 months in Kenya and by the number of delivery clients present on the day of the survey multiplied by 365 in Malawi.

†† Highest quintile contains facilities with five clinical staff or more per maternity bed in Kenya and three or more in Malawi.

**Figure 1:**
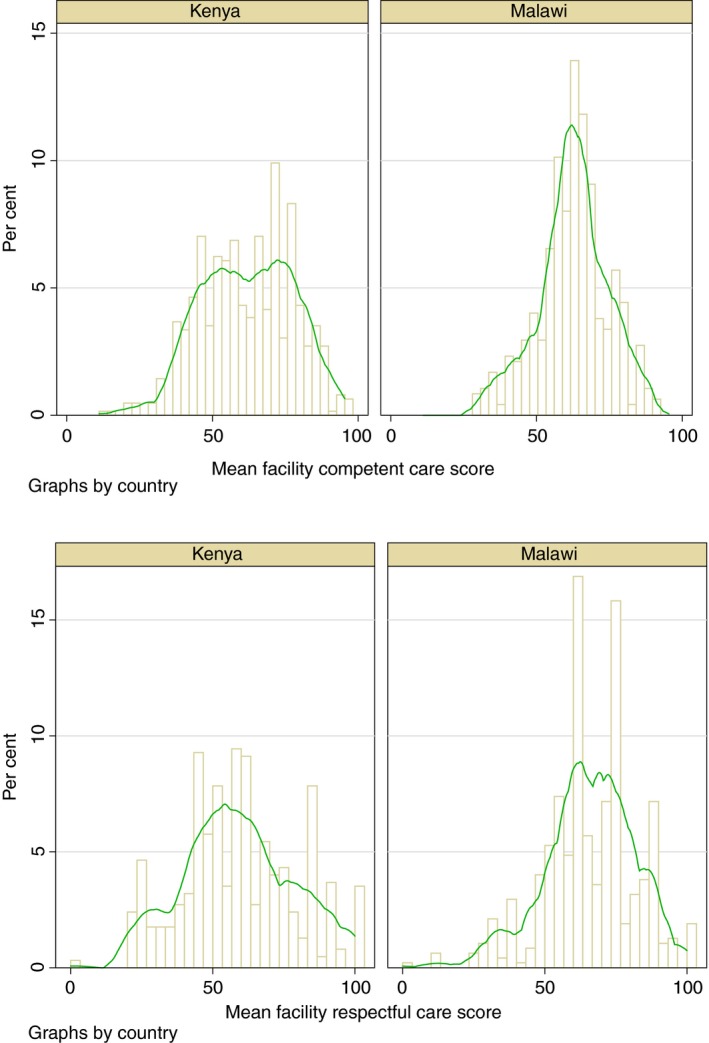
Distribution of competent and respectful care scores by facility and kernel density estimate plots in Kenya and Malawi.

**Figure 2:**
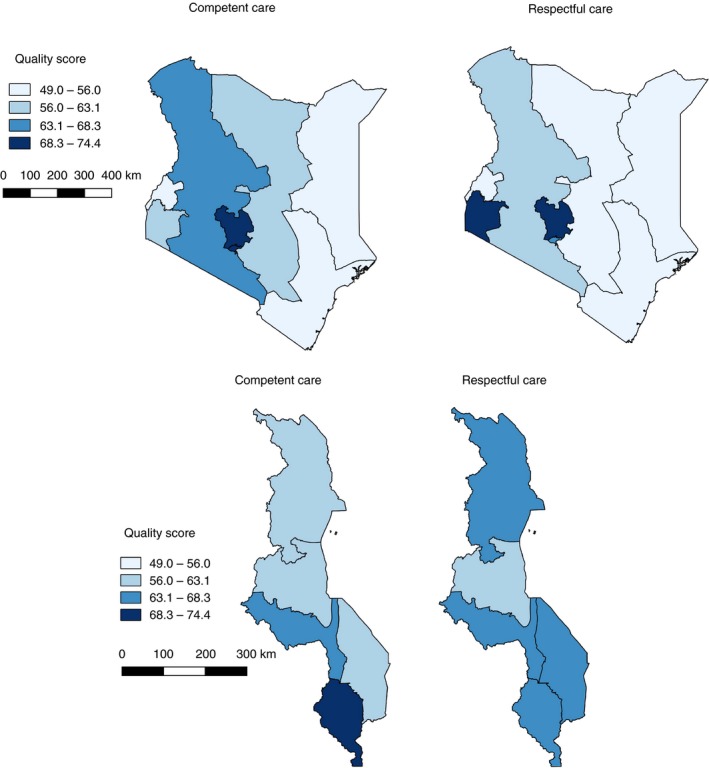
Geographic variation in competent and respectful labour and delivery care across Kenya and Malawi.

In Kenya, the score for average care competence was 62% and 61% for respectful care. In Malawi, the score for average care competence was 64% and 66% for respectful care. We found that variation in the scores was more pronounced across facilities in Kenya. Figure 1 shows that many facilities in Malawi had broadly similar scores. Mean quality scores and pairwise comparisons across levels of the covariates are shown in Tables S1 and S2. Quality scores were associated with several patient‐, provider‐ and facility‐level characteristics. For example, competent care was substantially higher in facilities performing more than 1500 deliveries per year and those with caesarean section capacity. In Figure S2, we plotted the mean (unadjusted) facility QoPIIPC index by the annual volume of deliveries across the 392 facilities from both countries. It shows that competent care tends to increase in higher‐volume facilities. Quality also varied substantially across regions in Kenya but did not meaningfully vary across zones in Malawi (Figure 2).

Tables 2 and 3 present the results of the fully adjusted, multivariable, random‐intercept regression models. In Kenya (Table 2), women delivering in the morning were significantly more likely to receive more competent and respectful care. Delivery in private hospitals was associated with a 13.7% increase in competent care (95% CI 4.95, 22.46) compared to public health centres. Public hospitals also appeared to provide more competent care than public health centres but the association was not significant. Better‐staffed facilities (i.e. those with the highest ratio of clinical providers per maternity bed) also provided more competent care. Using the Coast region as the reference, all region dummies (except for the Northeast and Nyanza) were significantly associated with competent care – with a 24.57% better score in Nairobi (95% CI 18.04, 31.09). Three regions also had higher respectful care scores (Nyanza, Central and Nairobi) than the Coast region. In adjusted models in Malawi (Table 3), the two younger age groups were associated with a 6.89% and 6.18% increase in competent care compared to women older than 35 (Table 3). Being HIV positive was also positively independently associated with competent care and primiparas received more respectful care. Delivering in a private hospital was associated with a 7.35% increase in competent care (95% CI 2.67, 12.03). Finally, facilities in the highest quintile of clinical providers per maternity bed had significantly higher competent (7.65, 95% CI 1.81, 13.49) and respectful care scores (11.36 95% CI 2.57, 20.16).

**Table 2:** Results of linear mixed models of observed competent and respectful labour and delivery care in Kenya, 2010 (standard errors clustered by facilities)

	Competent care (*N* = 622)			Respectful care (*N* = 598)		
	Coeff †	LCL‡	UCL	Coeff †	LCL‡	UCL
Patient						
Time of day						
Evening and night (6 pm–6 am)	*ref*			*ref*		
Afternoon (12 pm–5 pm)	2.51	−2.92	7.93	3.40	−2.32	9.12
Morning (7 am–12 pm)	5.00*	0.13	9.87	6.96**	2.10	11.82
Provider						
Gender						
Male	*ref*			*ref*		
Female	−1.65	−6.02	2.71	−3.34	−8.96	2.27
Cadre						
Lower §	*ref*			*ref*		
Higher¶	1.33	−2.10	4.76	2.36	−2.03	6.74
Facility						
Type						
Public health centre or dispensary	*ref*			*ref*		
Private health centre or maternity	0.92	−9.50	11.34	8.41	−6.79	23.60
Public hospital	5.14	−3.37	13.65	−2.10	−16.00	11.80
Private hospital	13.70**	4.95	22.46	12.88	−1.74	27.51
Clinical staff per maternity bed						
Less than 5	*ref*			*ref*		
5 or more	7.94**	2.52	13.37	4.59	−1.80	10.98
Regions						
Coast	*ref*			*ref*		
Central	23.87***	17.16	30.57	18.67***	9.39	27.95
Eastern	12.90**	3.74	22.05	4.72	−5.80	15.25
Nairobi	24.57***	18.04	31.09	17.86**	5.38	30.34
Northeastern	2.18	−5.11	9.47	0.73	−9.64	11.10
Nyanza	7.58	−0.95	16.11	24.55***	15.92	33.18
Rift Valley	14.25***	8.42	20.09	7.25	−3.44	17.95
Western	8.75**	2.75	14.74	3.03	−4.57	10.63
Intercept	38.10	27.67	48.54	43.25	27.79	58.70
Total variance	236.79			447.58		
Provider variance	110.82	77.70	158.06	151.47	101.74	225.50
Residual variance	125.97	95.00	167.05	296.11	235.00	373.12
Proportion of variance explained	32.9%			25.4%		

A total of 626 deliveries were observed in Kenya but 4 women were missing data on all 20 indicators to measure the competent care index and 28 were missing all nine indicators required to measure the respectful care index, leading to analytical samples of 622 and 598 women respectively for each outcome.

† The coefficient is the expected difference in visit quality (scale 0 to 100) given a 1 unit difference in the exposure, holding all other covariates constant.

‡ Lower and upper confidence limits.

§ Lower cadres include enrolled nurses and midwives, community health nurses and nurse aides.

¶ Higher cadres include MDs, clinical technicians, medical assistants, BScN nurses and midwives and registered nurses and midwives.

*P ≤ 0.05, **P ≤ 0.01, ***P ≤ 0.001.

**Table 3:** Results of linear mixed models of observed competent and respectful labour and delivery care in Malawi, 2013–2014 (standard errors clustered by facilities)

	Competent care (*N* = 474)			Respectful care (*N* = 473)		
	Coeff †	LCL‡	UCL	Coeff †	LCL‡	UCL
Patient						
Time of day						
Evening and night (18pm–6am)	*ref*			*ref*		
Afternoon (12pm–17pm)	0.31	−6.02	6.65	4.51	−3.13	12.14
Morning (7am–11am)	0.31	−5.99	6.61	4.20	−3.00	11.41
Age of the woman						
35+	*ref*			*ref*		
20–35	6.89***	2.52	11.27	5.24	−1.75	12.24
19 or less	6.18**	2.71	9.66	3.38	−4.60	11.36
First childbirth						
No	*ref*			*ref*		
Yes	1.39	−1.63	4.40	4.91*	0.20	9.62
HIV positive						
No	*ref*			*ref*		
Yes	4.48*	0.10	8.86	4.45	−2.05	10.96
Provider						
Gender						
Male	*ref*			*ref*		
Female	2.97	−0.83	6.77	4.00	−1.49	9.48
Cadre						
Lower§	*ref*			*ref*		
Higher¶	3.72	−0.05	7.49	−2.50	−8.17	3.17
Facility						
Location						
Rural	*ref*			*ref*		
Urban	3.59	−0.60	7.77	1.29	−4.92	7.51
Type						
Public health centre	*ref*			*ref*		
Private health centre, maternity or clinic	2.64	−2.11	7.40	−2.52	−8.72	3.68
Public hospital	0.54	−4.17	5.25	−6.03	−12.37	0.31
Private hospital	7.35**	2.67	12.03	0.19	−6.50	6.87
Clinical staff per maternity bed						
Less than 3	*ref*			*ref*		
3 or more	7.65**	1.81	13.49	11.36*	2.57	20.16
Zones						
Central east	*ref*			*ref*		
Central west	0.46	−4.64	5.57	1.19	−6.33	8.71
Northern	0.90	−4.44	6.24	0.65	−8.05	9.35
South east	2.02	−3.37	7.41	3.97	−3.87	11.81
South west	2.59	−2.56	7.74	0.94	−7.48	9.35
Intercept	48.49	39.55	57.44	52.51	40.39	64.64
Total variance	173.86			374.47		
Provider variance	80.66	54.21	120.00	105.14	61.79	178.93
Residual variance	93.20	69.92	124.24	269.33	213.84	339.21
Proportion of variance explained	13.8%			6.5%		

A total of 474 deliveries were observed in Malawi but one woman was missing all nine respectful care indicators, leading to analytical samples of 474 and 473 respectively for each outcome.

† The coefficient is the expected difference in visit quality (scale 0 to 100) given a 1 unit difference in the exposure, holding all other covariates constant.

‡ Lower and upper confidence limits

§ Lower cadres include enrolled nurses and midwives, community health nurses and nurse aides.

¶ Higher cadres include MDs, clinical technicians, medical assistants, BScN nurses and midwives and registered nurses and midwives.

*P ≤ 0.05, **P ≤ 0.01, ***P ≤ 0.001.

Overall in Kenya, the full model explained 33% of the variance in care competence and 25% of the variance in respectful care. Over 67% of the explained variance in both scores was due to the regions and around 30% to facility characteristics. Provider characteristics and time of delivery contributed little to the explained variance. In Malawi, the full model explained 14% of the variance in care competence and only 7% of the variance in respectful care. Most of the explained variance was due to facility and patient‐level characteristics. Zones and provider‐level covariates contributed little.

Our findings were largely unchanged in sensitivity analyses restricted to the 800 women with complete data, in regression analyses including sampling weights, and in the pooled model including data from both countries. Variance estimates and results from sensitivity analyses are available from the corresponding author.

## Discussion

In this study, we used data from observations of 1100 deliveries in two sub‐Saharan African countries to describe levels of competent and respectful delivery care and to investigate potential determinants of quality. Good quality care requires the provision of evidence‐based clinical actions and respectful care by providers. Our findings showed that labour and delivery care services are lacking in these two dimensions of quality: fewer than two‐thirds of basic elements of competent and respectful care were performed.

Several facility characteristics were associated with levels of competent and respectful care including facility ownership, type, volume and staffing. First, we found that private hospitals provided more competent care than other facility types. Private hospitals in sub‐Saharan Africa are generally located in capital cities and large urban areas, charge substantial user fees and are predominantly used by wealthier segments of the population. This disparity in quality of childbirth services between private hospitals and other facility types represents a major inequity. Public hospitals also appeared to perform better than health centres for competent care but not respectful care. Others have found that disrespect and abuse during childbirth is more common in public hospitals than in health centres or private facilities 7. In addition to having an intrinsic value, respectful, patient‐centred care is crucial to improve retention in care, adherence to treatments, and, ultimately, confidence in health systems 1. Public facilities must ensure that respectful provider attitude is prioritised along with improvements in care competence.

In univariate analyses, we also found evidence that care competence may be higher in higher‐volume facilities and those with caesarean section capacity. The effects of delivery volumes on quality of care have been studied in high‐income settings, and evidence indicates improved neonatal survival and fewer complications in higher‐volume facilities 13. In LMICs, studies have shown better maternity care quality, better inpatient care quality for small and sick newborns, and better maternal and newborn care provider knowledge in busier facilities compared to low case‐load facilities 14, 15, 16. The feasibility of relocating delivery services to higher‐volume facilities and those with surgical capacity (often hospitals), while ensuring equitable access for all women, should be studied in low‐income countries as it may be an effective approach to reduce maternal and newborn deaths. Future research is needed to develop and test context‐specific redesign strategies for improved childbirth care 17.

Care competence (in both countries) and respectful care (in Malawi) were also considerably higher in facilities with a higher ratio of clinical staff per maternity bed. Shortages of skilled clinical staff remain an important problem in sub‐Saharan Africa and appear to reduce providers ability to provide high‐quality care. In this study, virtually all deliveries observed were performed by nurses and midwives. Only 1% of observed deliveries in Kenya were performed by MDs and none in Malawi. According to the 2013 Kenyan service availability and readiness assessment survey, the doctor to population ratio is less than one (<1) per 10 000 population, the registered clinical officer ratio is 1.1 per 10 000, and the nurse population ratio is 3 per 10 000 18. These fall short of the recommended target of 23 healthcare professionals (counting physicians, nurses and midwives) per 10 000 2. Increasing numbers and skills of the health workforce is crucial to improve quality care in sub‐Saharan Africa 1, 19, 20. Staff shortages may also explain the lower levels of quality found in evening and night deliveries. Studies in the United States have shown that delivery complications tend to be higher during night shifts and on weekends and holidays, when hospitals are understaffed, and less experienced doctors are more likely to work 21. In low‐income countries, studies found that disrespectful and abusive experiences are more common, and women are more likely to be left alone during night shifts while there are fewer providers 22, 23. In both high‐ and low‐income countries, quality care and good health outcomes could be improved through strategic scheduling of staff.

In Kenya, we found that the regions were the strongest determinant of variation in quality. Major ethnic, cultural and economic differences exist across regions in Kenya and the region dummies may capture some of these unmeasured factors. For example, others have found that women in lower wealth quintiles and those living in multidimensionally poor areas tend to receive lower quality maternity care 24, 25. Kenya has had a long history of decentralisation dating back to the early 1980s, and differences in policies and resource allocation between regions may explain the variation in quality of care 26. In contrast, quality was fairly homogeneous across geographic subdivisions in Malawi which may reflect the effects of a fairly centralised health system, run within the capital. Nonetheless, health system financing and management is gradually being decentralised in Malawi 27. Efforts should focus on addressing these structural inequities and determinants of poor‐quality care.

Finally, we found that individual providers in Malawi appear to give different care to different women. Younger women and those giving birth for the first time received higher‐quality care than women aged over 35. This finding is particularly concerning given that older women are at higher risk for complications than the 20–35 age group 28. Providers may believe that older women and those with previous childbirths require less attention and support since they are presumed to be experienced in childbirth. These biases should be addressed during preservice education.

In the adjusted model, HIV‐positive women also received more competent care. When attending to HIV‐positive women, providers may be following clinical guidelines more closely. HIV‐positive women may also be benefiting from prevention of mother‐to‐child transmission (PMTCT) programs and giving birth in better‐funded or better‐staffed wards. The presence of donor‐funded HIV programs in a facility is associated with better quality care in areas unrelated to HIV 29, 30. While a patient’s specific presentation can alter providers’ clinical actions during delivery, the items included in the two quality indices we used represent basic procedures that should be done for all deliveries. Women should receive these basic levels of quality and respect during childbirth irrespective of age, gravidity and HIV status.

Our study analysed variation in quality of delivery care in Kenya and Malawi and identified opportunities for improving levels of competent and respectful care. To our knowledge, our study is the first to jointly assess care competence and respectful maternity care to give a more complete picture of quality. Nonetheless, our study has several limitations. First, the data used are several years old (2010 in Kenya and 2013–2014 in Malawi) and may not reflect current levels of quality. Since the survey, Kenya has also designated new subnational units – counties. Nonetheless, findings on variation and associated factors are still important to inform future policies. Second, although direct observation of care is the gold standard method to assess the quality of processes of care, it is susceptible to observer error and the Hawthorne effect; the latter would bias our results upward suggesting actual quality is worse than described here. In addition, because the delivery process is unpredictable, data on specific indicators were missing for several women who were only observed for certain portions of the delivery process. Nonetheless, our findings were largely unchanged when repeated in the subsample of women with complete data. This study did not assess the quality of processes of care during obstetric complications which are responsible for the majority of maternal and neonatal deaths. The SPA survey also does not include data on birth outcomes. Linking process quality to maternal and newborn morbidity and mortality would be valuable in future studies. Our study was also limited to covariates measured in the SPA surveys. Two‐thirds of the variation in quality in Kenya and 86% of the variation in care competence in Malawi remain unexplained. Other factors not included here likely affect quality, such as individual patient characteristics in Kenya, provider knowledge, skills, motivation, experience or remuneration and quality of facility management.

## Conclusion

It has now become evident that without attention to quality, universal health coverage will underdeliver on promised health gains 31. In 2018, three reports on the state of healthcare quality were published and concluded that new approaches were needed to close the global healthcare quality gap 32. Our analysis sheds light on the inadequate levels of labour and delivery care in sub‐Saharan Africa. High‐quality, respectful care during childbirth is a fundamental right of mothers and babies. Improvement efforts should focus on raising levels of quality and standardising care within countries. This may require substantial structural reforms in provider training, facility staffing levels, and policies about the overall competence and volume of facilities that should be providing labour and delivery care services. Learning what leadership and accountability approaches have been adopted by better performing regions will also be instructive.

## Supporting information


**Figure S1.** Indicators included in the competent care (quality of the process of intrapartum and immediate postpartum care (QoPIIPC)) and the respectful maternity care indices.
**Figure S2** **.** Average facility care competence (QoPIIPC index) by annual volume of delivery in Kenya and Malawi.
**Table S1** **.** Competent and respectful care scores across covariates, Kenya (2010).
**Table S2** **.** Competent and respectful care scores across covariates, Malawi (2013–2014).
