# Supplementary material for: Variation in competent and respectful delivery care in Kenya and Malawi: a retrospective analysis of national facility surveys
Source: Trop Med Int Health. 2020 Jan 2;25(4):442–53. doi: 10.1111/tmi.13361 (PMC7217001; doi:10.1111/tmi.13361)
Supplement: Supplementary file 1 — Figure S1. Indicators included in the competent care (quality of the process of intrapartum and immediate postpartum care (QoPIIPC)) and the respectful maternity care indices. Figure S2 . Average facility care competence (QoPIIPC index) by annual volume of delivery in Kenya and Malawi. Table S1 . Competent and respectful care scores across covariates, Kenya (2010). Table S2 . Competent and respectful care scores across covariates, Malawi (2013–2014). [file TMI-25-442-s001.docx]

**Supplementary figures and tables**

**Figure S1.** *Indicators included in the competent care (quality of the process of intrapartum and immediate postpartum care (QoPIIPC)) and the respectful maternity care indices*

| **Competent care – QoPIIPC index** | 1. Initial client assessment and examination | Checks woman's HIV status (checks chart or asks woman) and/or offers woman HIV test ^a^ |
| --- | --- | --- |
|  |  | Asks whether woman has experienced headaches or blurred vision ^a^ |
|  |  | Asks whether woman has experienced vaginal bleeding ^a^ |
|  |  | Asks about danger signs ^b^ |
|  |  | Takes blood pressure |
|  |  | Takes pulse |
|  |  | Washes his/her hand before any examination |
|  |  | Wears high-level disinfected or sterile gloves for vaginal examination |
|  | 2. First stage of labor |  |
|  |  | At least once, explains what will happen in labor to the woman and/or her support person |
|  |  | Prepares uterotonic drug to use for AMTSL |
|  |  | Uses partograph (during labor) |
|  |  | Self-inflating ventilation bag (500mL) and face masks (size 0 and size 1) are laid out and ready for use for neonatal resuscitation |
|  | 3. Second and third stages of labor |  |
|  |  | Correctly administers uterotonic (timing, dose, route) |
|  |  | Assesses completeness of placenta and membranes |
|  |  | Assesses for perineal and vaginal lacerations |
|  | 4. Immediate newborn and postpartum care |  |
|  |  | Immediately dries baby with towel |
|  |  | Places newborn on mother’s abdomen skin-to-skin |
|  |  | Ties or clamps cord when pulsations stop, or by 2–3 minutes after birth (not immediately after birth) |
|  |  | Takes mother's vital signs 15 minutes after birth |
|  |  | Palpates uterus 15 minutes after birth |
|  |  | Assists mother to initiate breastfeeding within one hour |
| **Respectful maternity care** | | Respectfully greets the pregnant woman |
|  |  | Encouraged client to have a support person present |
|  |  | Asks client if she has any questions |
|  |  | Explained procedures before proceeding |
|  |  | Informed client of findings |
|  |  | Encouraged food & fluids consumption during labor |
|  |  | Encouraged client to ambulate/change labor positions |
|  |  | Supports client in friendly way during labor |
|  |  | Drapes client before delivery |
| a Only collected in Malawi | | |
| b Only collected in Kenya | | |

| **Table S1.** *Competent and respectful care scores across covariates, Kenya (2010)* | | | | |
| --- | --- | --- | --- | --- |
|  | **Competent care** (N= 622) | | **Respectful care score** (N= 598) | |
|  | **mean ^a^** | **SD** | **mean ^a^** | **SD** |
| **Overall score** | 61.6 | 18.8 | 60.5 | 24.6 |
|  | **mean ^a^** | **p-value ^b^** | **mean ^a^** | **p-value ^b^** |
| **Patient (N= 626)** |  |  |  |  |
| Time of day |  |  |  |  |
| Evening and night (18pm-6am) | 58.2 | ref | 51.7 | ref |
| Afternoon (12pm-17pm) | 58.5 | 0.319 | 57.3 | 0.051 |
| Morning (7am-11am) | 64.2 | 0.000*** | 64.5 | 0.000*** |
| **Provider (N= 331)** |  |  |  |  |
| Gender |  |  |  |  |
| Male | 58.4 | ref | 63.7 | ref |
| Female | 62.3 | 0.169 | 59.9 | 0.891 |
| Cadre |  |  |  |  |
| Lower ^c^ | 58.5 | ref | 56.3 | ref |
| Higher ^d^ | 63.2 | 0.004** | 62.7 | 0.006** |
| **Facility (N= 170)** |  |  |  |  |
| Type |  |  |  |  |
| Public health center or dispensary | 51.5 | ref | 57.8 | ref |
| Private health center or maternity | 53.4 | 1.000 | 68.4 | 0.483 |
| Public hospital | 61.6 | 0.274 | 57.5 | 1.000 |
| Private hospital | 71.3 | 0.000*** | 71.4 | 0.040* |
| C-section capacity |  |  |  |  |
| No | 53.4 | ref | 60.5 | ref |
| Yes | 65.2 | 0.000*** | 60.5 | 0.972 |
| Annual volume of deliveries |  |  |  |  |
| < 500 | 54.9 | ref | 63.4 | ref |
| 500-1500 | 57.3 | 0.461 | 61.2 | 1.000 |
| 1500+ | 67.8 | 0.000*** | 58.6 | 0.551 |
| Clinical staff per maternity bed |  |  |  |  |
| Less than 5 | 57.7 | ref | 58.6 | ref |
| 5 or more | 72.5 | 0.000*** | 66.1 | 0.005** |
| **Regions (N= 8)** |  |  |  |  |
| Central | 75.4 | 0.000*** | 70.9 | 0.000*** |
| Nairobi | 72.6 | 0.000*** | 66.0 | 0.000*** |
| Rift Valley | 64.1 | 0.000*** | 58.7 | 1.000 |
| Eastern | 60.8 | 0.003** | 51.4 | 1.000 |
| Nyanza | 58.1 | 0.001** | 73.1 | 0.000*** |
| Western | 55.0 | 0.195 | 49.1 | 1.000 |
| Northeastern | 51.0 | 1.000 | 52.8 | 1.000 |
| Coast | 49.9 | ref | 50.4 | ref |
| a Includes patient-level sampling weights |  |  |  |  |
| b P-values derived from pairwise comparisons of means between each level of covariates and the reference category. Bonferonni method was used to adjust for multiple comparisons for categorical variables | | | | |
| c Lower cadres include enrolled nurses and midwives, community health nurses and nurse aides. | |  |  |  |
| d Higher cadres include MDs, clinical technicians, medical assistants, BScN nurses and midwives and registered nurses and midwives. | | | |  |
| *P ≤0.05, ** P ≤0.01, ***P ≤0.001. |  |  |  |  |

| **Table S2.** *Competent and respectful care scores across covariates, Malawi (2013-2014)* | | | | |
| --- | --- | --- | --- | --- |
|  | **Competent care** (N = 474) | | **Respectful care score** (N= 473) | |
|  | **mean ^a^** | **SD** | **mean ^a^** | **SD** |
| **Overall score** | 64.2 | 13.7 | 65.8 | 19.8 |
|  | **mean ^a^** | **p-value ^b^** | **mean ^a^** | **p-value ^b^** |
| **Patient (N= 474)** |  |  |  |  |
| Time of day Evening and night (18pm-6am) | 61.9 | ref | 61.7 | ref |
| Afternoon (12pm-17pm) | 64.6 | 1.000 | 64.1 | 1.000 |
| Morning (7am-11am) | 64.2 | 1.000 | 66.7 | 1.000 |
| Age of the woman 35+ | 58.3 | ref | 58.8 | ref |
| 20-35 | 64.6 | 0.034* | 66.7 | 0.143 |
| 19 or less | 66.2 | 0.012* | 66.6 | 0.074 |
| First childbirth |  |  |  |  |
| No | 63.2 | ref | 64.4 | ref |
| Yes | 66.5 | 0.005** | 68.8 | 0.016* |
| HIV positive |  |  |  |  |
| No | 64.0 | ref | 65.6 | ref |
| Yes | 67.0 | 0.038* | 67.9 | 0.312 |
| **Provider (N= 292)** |  |  |  |  |
| Gender |  |  |  |  |
| Male | 62.6 | ref | 63.7 | ref |
| Female | 64.6 | 0.018* | 66.3 | 0.099 |
| Cadre |  |  |  |  |
| Lower ^c^ | 63.1 | ref | 65.4 | ref |
| Higher ^d^ | 67.7 | 0.004** | 66.8 | 0.225 |
| **Facility (N= 222)** |  |  |  |  |
| Location Rural | 61.3 | ref | 65.7 | ref |
| Urban | 66.4 | 0.001** | 65.8 | 0.827 |
| Type |  |  |  |  |
| Public health center | 59.8 | ref | 66.9 | ref |
| Private health center, maternity or clinic | 60.5 | 1.000 | 63.6 | 1.000 |
| Public hospital | 65.7 | 0.015* | 65.1 | 0.833 |
| Private hospital | 67.0 | 0.001** | 67.9 | 1.000 |
| C-section capacity |  |  |  |  |
| No | 60.3 | ref | 65.5 | ref |
| Yes | 66.3 | 0.000*** | 65.9 | 0.953 |
| Annual volume of deliveries ^e^ |  |  |  |  |
| < 500 | 60.9 | ref | 65.6 | ref |
| 500-1500 | 63.3 | 1.000 | 65.7 | 1.000 |
| 1500+ | 65.2 | 0.119 | 65.8 | 1.000 |
| Clinical staff per maternity bed |  |  |  |  |
| Less than 3 | 62.5 | ref | 64.5 | ref |
| 3 or more | 72.3 | 0.000*** | 71.7 | 0.015* |
| **Zones (N= 5)** Central east | 62.5 | ref | 61.5 | ref |
| Central west | 64.1 | 1.000 | 66.4 | 1.000 |
| Northern | 62.9 | 1.000 | 66.0 | 1.000 |
| South east | 62.9 | 1.000 | 67.4 | 1.000 |
| South west | 68.7 | 0.943 | 67.8 | 1.000 |
| a Includes patient-level sampling weights |  |  |  |  |
| b P-values derived from pairwise comparisons of means between each level of covariates and the reference category. Bonferonni method was used to adjust for multiple comparisons for categorical variables | | | | |
| c Lower cadres include enrolled nurses and midwives, community health nurses and nurse aides. | | | | |
| d Higher cadres include MDs, clinical technicians, medical assistants, BScN nurses and midwives and registered nurses and midwives. | | | | |
| e Annual volume of deliveries was estimated by the number of delivery clients present on the day of the survey multiplied by 365 | | | | |
| *P ≤0.05, ** P ≤0.01, ***P ≤0.001. |  |  |  |  |

**Figure S2**. *Average facility care competence (QoPIIPC index) by annual volume of delivery in Kenya and Malawi*

Annual delivery volume and facility quality for observed (unweighted) sample of deliveries with loess smoother line shown for all health-care facilities ( n=392). Facilities are truncated at 15 000 deliveries per year (2 facilities excluded) to show the region of the plots with most facilities.
